# Predicting Trajectories of Everyday Functioning in Adults Aging with HIV Using Latent Growth Mixture Modeling

**DOI:** 10.1007/s10461-025-04623-z

**Published:** 2025-02-10

**Authors:** Lillian Ham, Scott Roesch, Donald R. Franklin, Ronald J. Ellis, Igor Grant, David J. Moore

**Affiliations:** 1 San Diego Joint Doctoral Program in Clinical Psychology, San Diego State University/University of California, San Diego, CA USA; 2 HIV Neurobehavioral Research Program, University of California San Diego, 220 Dickinson Street, Suite B (8231), San Diego, CA 92103 USA; 3 Department of Psychology, San Diego State University, San Diego, CA USA; 4 Department of Psychiatry, University of California San Diego, La Jolla, CA USA; 5 Department of Neurosciences, University of California San Diego, La Jolla, CA USA

**Keywords:** Activities of daily living, Cognition, Neuropsychology, Latent variable modeling, Motor skills, Depression

## Abstract

As the U.S. population of people with HIV (PWH) ages, PWH exhibit high rates of adverse health outcomes including everyday functioning decline. We aimed to (1) identify trajectories of self-reported everyday functioning and (2) examine baseline predictors (demographics, cognitive domains, psychiatric and medical comorbidities, HIV-disease characteristics) of trajectories among PWH. 742 PWH completed up to five semi-annual visits over two years. Latent growth mixture modeling identified a linear 3-class solution with good statistical fit and interpretability. Most PWH (88%) had good baseline functioning with stability. Two classes had elevated baseline functional declines with worsening (7%) or improvement (5%). Greater depressive symptoms and motor skills impairment predicted higher odds of impaired functioning. Having chronic pulmonary disease increased odds of improvement, which may reflect connection to care, while older age increased odds of worsening. Most aging PWH demonstrate stable everyday functioning; however, interventions for depression and motor skills may improve functioning.

## Resumen

A medida que la población de individuos con VIH en Estados Unidos envejece, sus riesgos de salud aumentan, incluyendo la capacidad de realizar actividades de la vida diaria (AVD). Nuestros objetivos fueron (1) identificar trayectorias de capacidad para realizar AVD en individuos con VIH y (2) examinar características (información demográfica, capacidad cognitiva, enfermedades y características de VIH) que puedan predecir estas trayectorias. 742 participantes con VIH completaron hasta cinco visitas semestrales durante 2 años. Un modelo de curva de crecimiento mixto latente identificó una solución lineal de 3 clases con buen ajuste estadístico e interpretabilidad. En la mayoría de los participantes (88%) se observó buen y estable funcionamiento diario al principio del estudio. En dos clases (grupos) se encontraron peores disminuciones funcionales con deterioros (7%) o mejoras (5%) subsecuentes. Mayores síntomas depresivos y problemas motrices predijeron mayor probabilidad de impedimentos en AVD. Presencia de enfermedad pulmonar crónica predijo mejor capacidad de realizar AVD, un resultado que quizas refleja acceso a atención médica. Mayor edad predijo peor capacidad para realizar AVD. La mayoría de la gente envejeciendo con VIH demostró estabilidad en su capacidad de realizar actividades cotidianas. Intervenciones para la depresión y capacidad motriz podrían mejorar el funcionamiento diario.

## Introduction

There is significant evidence that people with HIV (PWH) experience a disproportionate burden of disease with older age [1] including neurocognitive impairment (NCI) [2, 3], and psychiatric [4, 5] and medical [1, 6] comorbidities. Greater comorbidity burden is linked to atypical aging processes [7–10], which is increasingly relevant as half of PWH in the U.S. are entering older adulthood [11]. Consequently, HIV is a chronic disease in which adverse health outcomes (e.g., functional decline) reflect cumulative, multi-system dysfunction [12].

HIV is often associated with impairments in everyday functioning [13, 14]. Functional impairment caused by underlying pathology may ultimately lead to disability [13]. Periods of disability may fluctuate across time, span beyond physical domains (e.g., social inclusion), and be exacerbated or alleviated by contextual factors (e.g., social support) [15]. Acquired functional declines have traditionally been defined by losses in the ability to independently manage one’s activities of daily living (ADL) [16–18] with often more complex or instrumental ADLs (IADL; e.g., finances) affected before basic ADLs (BADL; e.g., dressing).

### Predictors of Everyday Functioning

Among PWH, NCI, depression, and HIV-disease characteristics (e.g., nadir CD4) have been related to everyday functioning impairment [16, 19]. NCI is strongly associated with poorer everyday functioning (e.g., medication management, driving) [14, 17, 20, 21] and unemployment in PWH [14, 17, 22–24], particularly among older persons [25, 26]. Cross-sectional and longitudinal studies of PWH [14, 17, 20, 27–29] have linked motor skills, memory, and executive functioning to everyday functioning, which are domains commonly affected in HIV [30, 31]. Thus, characterizing the severity of impairment in everyday functioning has been critical to diagnosing and staging NCI, most commonly by Frascati criteria for HIV-associated neurocognitive disorders (HAND) [32]. Likewise, depression is not only highly prevalent among PWH [33–35], but also linked to greater functional difficulties [17, 19, 22, 36, 37] possibly via poor health behaviors [38–41]. A recent study [16] found that PWH were nearly five times more likely than people without HIV (PWoH) to experience major functional declines, and among PWH with NCI, about half exhibited at least mild functional decline.

Despite increases in ART adherence and viral suppression in older age [1], it is well supported that PWH experience increased comorbidity burden [42–45]. In the CNS HIV Antiretroviral Therapy Effects Research (CHARTER) study, the severity of clinician-classified comorbidity burden (i.e., incidental, contributing, confounding) [32] (see online supplement) has been incrementally related to greater functional decline, higher rates of unemployment, and NCI [46]. Confounding comorbidity burden was also predictive of neurocognitive decline over three years, in which 67% of neurocognitive decliners experienced functional decline [47]. Functional decline may even be better explained by comorbidities rather than age or HIV itself. One study [48] found that medical and psychiatric comorbidities predicted poorer physical functioning above and beyond age and HIV-disease characteristics (e.g., viral load). Based on findings in the literature thus far, measures of domain-specific cognitive impairment (e.g., motor skills), HIV-disease characteristics (e.g., nadir CD4), and medical (e.g., hepatitis C) and psychiatric comorbidities (e.g., major depressive disorder [MDD]) were considered as predictors of everyday functioning in the current study.

### Longitudinal Studies of Everyday Functioning

Many large, U.S. studies have identified distinct everyday functioning trajectories over several years among older PWoH. Prior work has identified at least three classes of trajectories with varying levels of baseline functioning and rates of decline, whereby the largest class is comprised of individuals with no disability or late decline (40–76% of samples) and the smallest class of individuals with rapid or large decline (8–21% of samples) [49–55]. Consistently, studies have found that age, cognition, and medical comorbidities differentiate functional trajectories [52, 56–58], whereas there are mixed findings for sociodemographic factors such as sex, race, and education [51, 53, 54, 59, 60].

The combined effects of age and HIV have been observed in cross-sectional studies with varying support. One study [19] found synergistic effects such that older PWH demonstrated the poorest everyday functioning in BADLs, IADLs, and clinician-rated functional impairment (i.e., Karnofsky Performance Status scale) compared to younger PWoH. Less compelling evidence has been observed in other cross-sectional studies. Two studies by Vance and colleagues observed no age by HIV interactions on performance-based measures of everyday functioning; however, trends suggested that older PWH are more prone to impaired functioning reflected by poorer neurocognitive performance [61] and subtle functional inefficiencies [62–64]. In the only found longitudinal study [65] to examine the effects of age and HIV on everyday functioning, older PWH exhibited stable, but high rates of functional decline over one year compared to younger PWH who showed modest functional decline and improvement at follow-up.

### Current Study Objectives

Evidence for the combined effects of age and HIV on everyday functioning has been derived almost entirely from cross-sectional observations without consideration of change in everyday functioning across time. Longitudinal study would allow for the detailed examination of everyday functioning trajectories and meaningful predictors of these trajectories. This study aimed to fill this gap, providing more nuanced insights that may inform targeted interventions for subgroups of this population. As such, our objectives were to: (1) identify unique everyday functioning trajectories among PWH, and (2) determine which demographics, cognitive domains, psychiatric and medical comorbidities, and HIV-disease characteristics predict classification into everyday functioning trajectories.

## Methods

PWH enrolled in the CHARTER program from 2003 to 2020 were considered for inclusion in the current study. Participants completed everyday functioning questionnaires, a blood draw, and comprehensive neuropsychological, neuromedical, and neuropsychiatric evaluations. HIV serostatus was determined by enzyme linked immunosorbent assay (ELISA) with a confirmatory Western Blot. Diagnoses of hypertension, hyperlipidemia, diabetes, and hepatitis C were based on self-report and/or the participant being on a medication respective to each condition at their visit. Hepatitis C diagnosis was also based on a positive antibody test. Chronic pulmonary disease (CPD) included either chronic obstructive pulmonary disease or asthma. Participants were drawn from six sites: Johns Hopkins University, Icahn School of Medicine at Mount Sinai, UC San Diego, University of Texas Medical Branch, University of Washington, and Washington University in St. Louis. All study procedures were approved by local Institutional Review Boards (IRB), and all participants provided written informed consent.

### Inclusion and Exclusion Criteria

Inclusion criteria were broad and did not exclude by comorbid conditions that may impact CNS function. CHARTER recruited participants that broadly reflect the geographic and sociodemographic diversity of PWH at university-affiliated treatment centers across the U.S. Medical history of participants was reviewed by two CHARTER clinician investigators and independently rated to determine the extent to which non-HIV-related comorbidities confounded the interpretation of neurocognitive and everyday functioning, integral to HAND classification. Participants with severely “confounding” comorbidities were excluded, which included individuals with conditions (e.g., neurodevelopmental, medical, psychiatric) that likely account for neurocognitive and/or everyday functioning impairments and preclude a diagnosis of HAND (i.e., deficits attributable to HIV). This approach has been supported by previous research showing that PWH with confounding comorbidities had overall worse brain integrity; however, those with contributing and incidental comorbidities had comparable brain abnormalities [66]. Exclusion criteria included history of a severe learning disability, psychotic disorder (e.g., schizophrenia), major neurological condition that may impair neurocognitive functioning (e.g., stroke), and evidence of intoxication by illicit substances (except cannabis) via positive urine toxicology screen or Breathalyzer test for alcohol on the day of testing.

### Measures

#### Everyday Functioning

Functional declines were assessed by the modified Instrumental Activities of Daily Living (IADL) [17, 67] questionnaire, which measured self-reported levels of independent functioning in 13 domains (housekeeping, managing finances, buying groceries, cooking, planning social activities, understanding read materials/TV, transportation, using the telephone, home repairs, shopping, laundry, medication management, work). Participants reported two ratings per IADL domain: current (“Now”) vs. best functioning level (“Best”). Any declines from “Best” to “Now” were counted as a functional “decline” with higher count scores representing greater functional dependence (total count range: 0–13). The modified IADL questionnaire consisted of an additional item asking if difficulties on everyday tasks were due to “primarily cognitive problems (e.g., thinking, memory, paying attention)”, “primarily physical problems (e.g., fatigue, feeling sick)”, or “equally cognitive and physical problems.” Responses endorsing primarily cognitive or equally cognitive and physical problems were coded as “attributed difficulties to cognitive problems”. The number of functional declines on this modified questionnaire was selected as the primary outcome measure as it has most commonly been used to determine functional dependence (i.e., ≥ 2 declines) in the context of HAND [16, 18, 46].

Two other measures of everyday functioning, which have also been used in the HIV literature [18, 68], were administered to validate and compare findings with the IADL questionnaire. The Patient’s Assessment of Own Functioning Inventory (PAOFI) [69] measured self-reported cognitive symptoms in daily life across 34 items (e.g., memory, language and communication, sensory-perceptual, higher level cognitive functions). Items measured the frequency of impairment in a domain with Likert-type responses (“Almost always” to “Almost never”). Responses occurring with at least “Fairly often” or greater frequency counted as a cognitive “complaint”. Higher count scores represented greater cognitive symptoms (total count range: 0–34).

The Karnofsky Performance Status (KPS) [70] scale measured participants’ ability to perform everyday tasks. A nurse rated participants’ overall functioning on a scale from 0 (dead) to 100 (normal, no complaints, no evidence of disease) with higher scores indicating better functioning.

#### Neuropsychological Assessment

Participants completed a standardized battery of 15 neuropsychological tests designed to provide a comprehensive assessment of seven cognitive domains most affected by HIV (Table 1): verbal fluency, executive functioning, processing speed, learning, memory, attention/working memory, and fine motor skills. To address potential confounds of demographic factors on cognition, individual raw scores were converted into demographically-adjusted (age, sex, education, race) T-scores, which were averaged within each domain and across the entire battery to derive domain-specific and global cognition T-scores, respectively.

**Table 1:** Neuropsychological battery

Neurocognitive Domain	Test
**Verbal fluency**	COWAT [71] Category Fluency (Animals); Letter Fluency (FAS)
**Executive functioning**	Wisconsin Card Sorting Test-64 (Perseverative responses) [71, 72] Trail Making Test, Part B [71]
**Processing speed**	WAIS-III [73] Digit Symbol Coding; Symbol Search Trail Making Test, Part A [71]
**Learning**	Hopkins Verbal Learning Test-Revised [72, 74] (Trials 1–3) Brief Visuospatial Memory Test-Revised [72, 75] (Trials 1–3)
**Memory**	Hopkins Verbal Learning Test-Revised [72, 74] (Delayed recall) Brief Visuospatial Memory Test-Revised [72, 75] (Delayed recall)
**Attention/working memory**	WAIS-III Letter-Number Sequencing [73] PASAT [76] (first channel only)
**Motor skills**	Grooved Pegboard Test [71, 77] (Dominant hand, non-dominant hand)

Note. COWAT = Controlled Oral Word Association Test, WASI-III = Wechsler Adult Intelligence Scale-3rd edition, PASAT = Paced Auditory Serial Addition Task

To determine how NCI relates to functional decline, the global deficit score (GDS) approach [78] was utilized. For greater specificity and focus on impairment, domain deficit scores (DDS) were considered as neurocognitive predictors in regression models. The GDS method assigns a deficit score to each T-score (M = 50, SD = 10), which increases with greater impairment (i.e., 0 = no cognitive impairment [T-scores ≥ 40]; 5 = severe cognitive impairment [T-scores < 20]). Deficit scores across all tests were averaged with a cut-off of GDS ≥ 0.5 indicating NCI. Deficit scores were averaged within cognitive domains to create DDS with a cut-off of DDS > 0.5 indicating NCI.

In post-hoc analyses, summary regression-based change scores (sRCS) were calculated to determine how changes in cognition over time related to changes in everyday functioning. The sRCS were calculated according to published guidelines that used normative data from PWH and PWoH [79]. The sRCS is a continuous z-score based on differences from observed versus predicted performance at the follow-up visit averaged across all 15 neuropsychological tests. The sRCS accounts for practice effects, regression towards the mean, and other factors that can influence test-retest variability in neurologically stable people (e.g., test-retest interval, demographics, overall neuropsychological performance at baseline). Two global z-scores were calculated: (1) a global total sRCS (i.e., the cumulative sum of all previous sRCS), and (2) a global average sRCS (i.e., global total sRCS / [total visit count– 1]). Higher z-scores indicated improvement in cognition over time.

#### Depressive Symptoms and Psychiatric Diagnoses

Depressive symptoms were measured by the Beck Depression Inventory-II (BDI-II) [80], a 21-item self-report questionnaire that measures the severity of depressive symptomatology in the last two weeks with higher scores reflecting greater depression (total sum range: 0–63). Diagnoses of current and lifetime MDD and substance use disorder (SUD) were assessed via the Composite International Diagnostic Interview (CIDI) [81], which follows DSM-IV diagnostic criteria.

### Statistical Analysis Plan

#### Identifying Everyday Functioning Trajectories

Latent growth mixture modeling (LGMM) was utilized to identify subpopulations with similar longitudinal trajectories. LGMM models both linear and non-linear trajectories and allows for individuals to vary on growth factors (i.e., intercept and slope) [82, 83]; thus, is appropriate for modeling everyday functioning, which we expected to vary between individuals and across time. Count IADL scores at each timepoint were used to create a latent slope variable to model everyday functioning trajectories and a latent intercept variable to represent scores at baseline. LGMM used maximum likelihood estimation with robust standard errors (MLR) to obtain optimal parameter estimates through an iterative process (i.e., EM algorithm). To address potential biases, full information likelihood (FIML) was used to minimize the impact of missing data [84]. FIML handles missing data by estimating a likelihood function for each participant based on observed variables so that all available data are used.

A series of models were conducted to determine the best-fitting solution (i.e., slopes, number of classes). First, baseline single-class latent growth curve models (LGCM) comparing linear and quadratic slopes were conducted. After, unconditional LGMMs without external predictors iteratively compared 1 to 4-class models. The best-fitting solution was determined based on statistical (i.e., information criteria statistics, entropy values, likelihood ratio tests) and theoretical criteria, including adequate class size (approximately ≥ 5% of the sample) and interpretability of classes. LGMM analyses were conducted in *Mplus* Version 8.10.

#### Predicting Class Membership

Multinomial logistic regression was conducted to identify the strongest predictors of classification into everyday functioning trajectories. Baseline predictors that showed significant omnibus differences by class membership were entered in the model. Differences in class membership were examined by one-way ANOVAs for continuous outcomes and chi-square tests of independence or Fisher’s exact tests for categorical outcomes. Non-parametric one-way Kruskal-Wallis tests were conducted for non-normally distributed outcomes. Significant omnibus tests were followed up by pairwise comparisons corrected for multiple comparisons using the Benjamini-Hochberg method.

Candidate predictors included: (1) demographics (e.g., age, gender, education, race), (2) medical and psychiatric comorbidities (e.g., CPD, lifetime MDD), (3) HIV-disease characteristics (e.g., nadir CD4, current CD4, viral load), and (4) cognition (e.g., motor deficit score). Regarding psychiatric factors, current MDD was excluded from the multinomial regression due to theoretical overlap with the BDI-II. The PAOFI and KPS were also excluded from the multinomial regression as they are alternate measures of everyday functioning and redundant with the outcome. Backwards stepwise selection was used to select variables for the final prediction model until all remaining variables were *p* < 0.20 [85]. To address potential confounds of multicollinearity [86], correlation coefficients (*r* ~ 0.8 indicating multicollinearity) and the variation inflation factor (VIF; VIF > 5 indicating multicollinearity) were examined. Regressions were conducted in JMP Pro 18.0.1 using two-tailed tests ($\:\alpha\:$ = 0.05).

## Results

### Participants and Missing data

Table 2 shows sample characteristics at baseline for all 742 participants who had IADL data. At baseline, 68.7% of participants had at least 1 IADL decline and 41.8% of participants had at least 2 IADL declines. Overall, 38.0% of participants attributed functional declines to primarily cognitive versus physical problems. Attrition rates over five visits across two years were as follows: 6 months: 17.1% (*N* = 615), 12 months: 44.7% (*N* = 410), 18 months: 51.1% (*N* = 363), and 24 months: 58.8% (*N* = 306). Variables associated with missingness were determined by calculating the cumulative number of missing visits per participant across all visits. Greater missing visits was associated with younger age (Spearman’s rho = -0.09, *p* = 0.01), fewer years of education (rho = -0.10, *p* < 0.01), fewer years of HIV disease (rho = -0.07, *p* = 0.04), being female (median = 2 vs. 1, Z = 2.24, *p* = 0.03), and having no hypertension (median = 2 vs. 1, Z = -2.61, *p* < 0.01), hyperlipidemia (median = 2 vs. 0, Z = -3.98, *p* < 0.01), and diabetes (median = 2 vs. 0, Z = -2.79, *p* < 0.01).

**Table 2:** Total sample characteristics at baseline

	CHARTER (*N* = 742)
*Demographics*	
Age (years)	43.1 (8.5)
Sex, N (% male)	575 (77.5)
Race/ethnicity, N (%)	
White (non-Hispanic)	320 (43.1)
Hispanic	81 (10.9)
Black	322 (43.4)
Asian	1 (0.1)
Other	18 (2.4)
Education (years)	13.0 (2.6)
WRAT-4 Reading standard score, M (SD)	93.9 (14.8)
Employed, N (%)	238 (34.3)
*Comorbidities*	
Hepatitis C, N (%)	176 (23.7)
Hypertension, N (%)	122 (16.4)
Hyperlipidemia, N (%)	61 (8.2)
Diabetes, N (%)	58 (7.8)
CPD, N (%)	62 (8.4)
Current smoker, N (%)	242 (32.7)
Past smoker, N (%)	571 (77.1)
*Psychiatric characteristics*	
Depressive sx (BDI-II)	11 [5–20]
Current MDD, N (%)	103 (14.0)
LT MDD, N (%)	370 (50.0)
Current substance use dx, N (%)	56 (7.6)
LT substance use dx, N (%)	529 (71.6)
*HIV characteristics*	
Current CD4 (c/µL)	437 [276–628]
Nadir CD4 (c/µL)	179 [50-312.3]
HIV duration (years)	9.7 [3.9–14.7]
On ART, N (%)	521 (70.2)
AIDS status, N (% AIDS)	303 (40.8)
Plasma viral load, N (% ≤ 50 c/µL)	325 (44.5)
*Neurocognition*	
Neurocognitive impairment, N (%)	231 (31.1)
Global deficit score, mdn [IQR]	0.3 [0.1–0.6]
Verbal fluency impairment, N (%)	107 (14.4)
Executive function impairment, N (%)	162 (21.8)
Processing speed impairment, N (%)	128 (17.3)
Learning impairment, N (%)	264 (35.6)
Memory impairment, N (%)	206 (27.8)
Working memory impairment, N (%)	176 (23.7)
Motor skills impairment, N (%)	139 (18.9)
*Daily functioning*,* mdn [IQR]*	
IADL declines	1 [0–3]
PAOFI complaints	2 [0–7]
KPS scale	90 [80–100]

Note. CHARTER = CNS HIV Antiretroviral Therapy Effects Research, CPD = chronic pulmonary disease, BDI-II = Beck Depression Inventory-II, sx = symptoms, dx = diagnosis, LT = lifetime, MDD = major depressive disorder, ART = antiretroviral therapy, IADL = independent activities of daily living, PAOFI = Patient’s Assessment of Functioning Inventory, KPS = Karnofsky Performance Status. M = mean, SD = standard deviation, mdn = median, IQR = interquartile range

### Optimal Latent Trajectory Class Solution

Table 3 presents the goodness-of-fit statistics for 1 to 4-class solutions, including Akaike information criteria (AIC), Bayesian information criteria (BIC), sample size-adjusted BIC (ssBIC), chi-square test, root mean square error of approximation (RMSEA), comparative fit index (CFI), entropy, and likelihood ratio tests (LRT). Fit statistics were excellent and comparable between baseline linear and quadratic LGCMs; thus, both linear and quadratic LGMMs were compared in 2 to 4-class solutions. AIC, BIC, and ssBIC statistics decreased with a greater number of classes, but entropy (i.e., class separation) was comparable between 2 to 3-class solutions. All three LRTs (Vuong-Lo-Mendell-Rubin [VLMR], Lo-Mendell-Rubin [LMR], parametric bootstrap [BLRT]) were significant for linear and quadratic 2-class solutions, suggesting that these models demonstrated improved fit relative to their 1-class solution. The linear 3-class solution also demonstrated a significant improvement from the linear 2-class solution; however, the quadratic 3-class solution did not indicate improvement from the quadratic 2-class solution on 2 of 3 LRTs. Thus, 2-classes were determined as optimal for the quadratic model. The 4-class linear solution did not demonstrate improvement from the 3-class linear solution on 2 of 3 LRTs and had poorer entropy; thus, 3-classes were determined as optimal for the linear model.

**Table 3:** Model fit statistics for baseline growth model and 2 to 4-class growth mixture models

	1-Class Baseline (Linear)	1-Class Baseline (Quadratic)	2-Class (Linear)	2-Class (Quadratic)	3-Class (Linear)^b^	3-Class (Quadratic)^b^	4-Class (Linear)^b^
**Sample size** ^a^							
N_c=1_	742	742	61	65	34	36	32
N_c=2_	--	--	681	677	49	39	39
N_c=3_	--	--	--	--	659	667	44
N_c=4_	--	--	--	--	--	--	627
**Fit statistics**							
# of Parameters	10	14	13	18	14	19	17
AIC	9987.0	9962.7	9662.0	9626.6	9551.3	9462.4	9508.1
BIC	10033.1	10027.2	9721.9	9709.5	9615.8	9550.0	9586.5
ssBIC	10001.4	9982.7	9680.7	9652.4	9571.4	9489.7	9532.5
Chi-square p-value	< 0.01	0.16	--	--	--	--	--
RMSEA (< 0.08)	0.05	0.03	--	--	--	--	--
CFI (> 0.90)	0.96	0.99	--	--	--	--	--
Entropy (> 0.80)	--	--	0.93	0.93	0.92	0.92	0.86
VLMR LRT p-value	--	--	< 0.01	< 0.01	< 0.01	0.38	0.54
LMR LRT p-value	--	--	< 0.01	< 0.01	< 0.01	0.39	0.55
BLRT p-value	--	--	< 0.01	< 0.01	< 0.01	< 0.01	< 0.01

Note. Model fit statistics and class sample sizes for 1 to 4-class solutions support that the 3-class linear solution is the best-fitting solution. Estimator for all models = maximum likelihood estimation with robust standard errors (MLR). a = estimated counts for classes based on most likely class membership, b = fixed linear slope variance to 0, c = class. Cut-offs in parentheses for fit statistics represent direction of optimal values. AIC = Akaike information criteria, BIC = Bayesian information criteria, ssBIC = sample size-adjusted BIC, RMSEA = root mean square error of approximation, CFI = comparative fit index; LRT = likelihood ratio test, VLMR = Vuong-Lo-Mendell-Rubin, LMR = Lo-Mendell-Rubin, BLRT = parametric bootstrap LRT

**Fig. 1:**
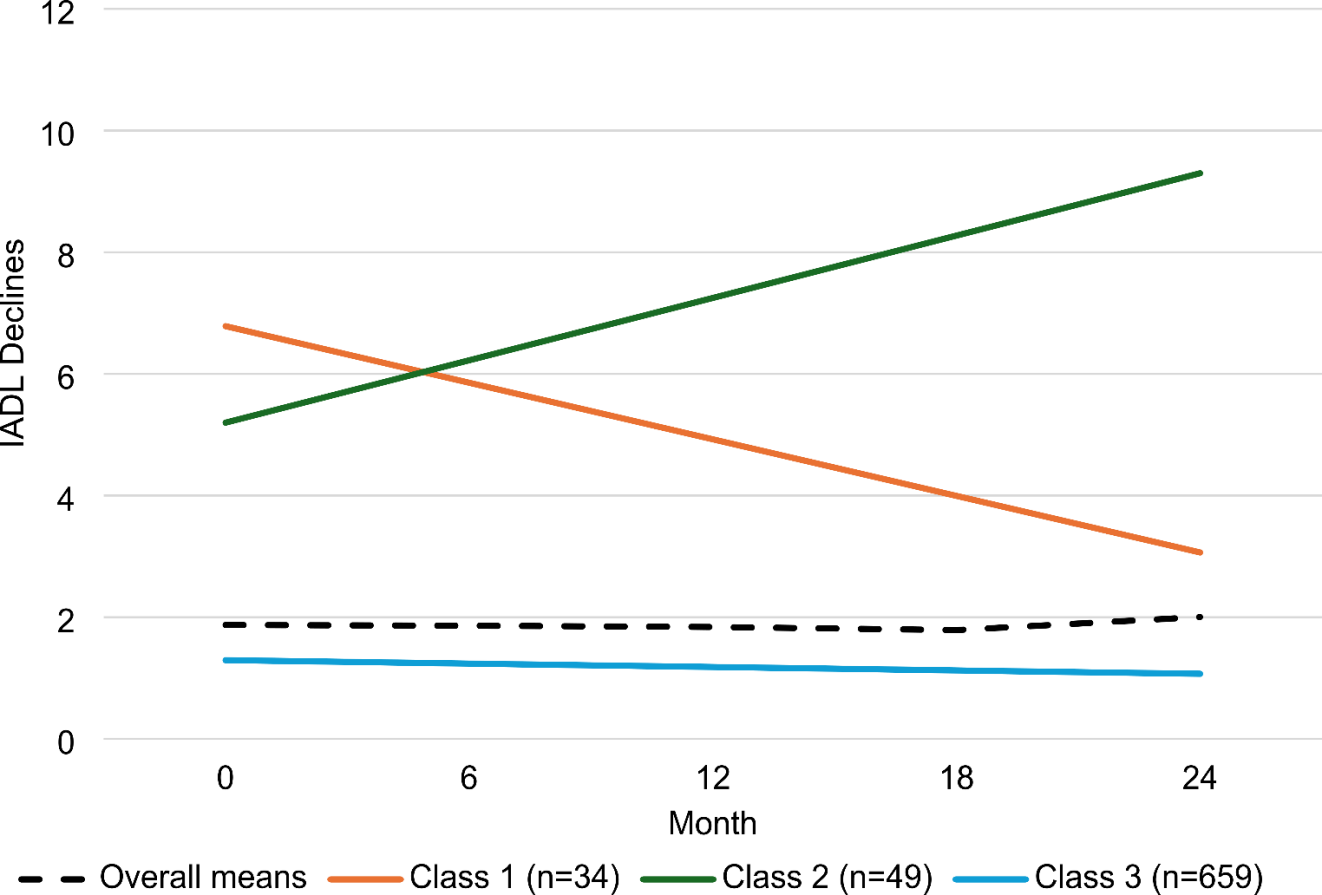
Trajectories of everyday functioning classes identified through a 3-class linear solution: Class 1 = Impaired & Improving (solid orange line), Class 2 = Impaired & Worsening (solid green line), and Class 3 = Unimpaired & Stable (solid blue line). Dotted black line = overall sample IADL means, IADL = Instrumental Activities of Daily Living

In the final comparison between the 3-class linear solution and the 2-class quadratic solution, both statistical fit indices and interpretability (e.g., class size) were considered. Though the smallest class size for the 3-class linear solution was less optimal (*n* = 34; 4.6% of total sample) than the 2-class quadratic solution (*n* = 65; 8.8% of total sample), entropy was comparable and the AIC, BIC, and ssBIC statistics were lower in the 3-class linear solution. Moreover, the 2-class quadratic solution did not have significant slopes for either class, whereas the 3-class linear solution did for all classes. Thus, the linear 3-class solution was determined as the best fitting model. The three longitudinal classes identified were (Fig. 1): Class 1 “Impaired and Improving” (4.6% of total sample; intercept[SE] = 6.8[0.7], *p* < 0.01; linear[SE] = -0.9[0.2], *p* < 0.01), Class 2 “Impaired and Worsening” (6.6% of total sample; intercept[SE] = 5.2[0.5], *p* < 0.01; linear[SE] = 1.0[0.2], *p* < 0.01), and Class 3 “Unimpaired and Stable” (88.8%; intercept[SE] = 1.3[0.1], *p* < 0.01; linear[SE] = -0.1[0.02], *p* < 0.01).

### Baseline Predictors of Trajectory Class Membership

Table 4 shows differences between classes on baseline characteristics with adjusted pairwise comparisons. Consistent with patterns of IADL declines, PAOFI complaints and KPS ratings were most impaired in Class 1_Impaired&Improving_ and Class 2_Impaired&Worsening_ versus Class 3_Unimpaired&Stable_; however, only IADL declines differed between Class 1_Impaired&Improving_ and Class 2_Impaired&Worsening_. Classes also differed in the attribution of functional declines to cognitive problems ($\:{\chi\:}^{2}$ = 44.2, *p * < 0.01): 79% of Class 1_Impaired&Improving_ and 65% of Class 2_Impaired&Worsening_ versus 34% of Class 3_Unimpaired&Stable_. All other baseline variables that significantly differed by class (Table 4) were considered as covariates in the multinomial regression. Regarding multicollinearity, Spearman’s rho correlations showed that the relationship between age and depressive symptoms (rho = 0.03), and age and motor deficit score (rho = 0.06) were weak and nonsignificant (*p*-values > 0.12). The correlation between depressive symptoms and motor deficit score was weak (rho = 0.15), but statistically significant (*p* < 0.01). VIFs for all predictors considered in the regression model were < 2.0 and non-suggestive of multicollinearity.

**Table 4:** Class differences by characteristics at baseline

	Class 1 Impaired & Improving (*n* = 34)	Class 2 Impaired & Worsening (*n* = 49)	Class 3 Unimpaired & Stable (*n* = 659)	Omnibus *p*-value	Sig. pairwise comparison^a^
*Demographics*					
Age (years)	45.3 (7.7)	46.7 (8.2)	42.7 (8.5)	< 0.01	2 > 3
Sex, N (% male)	26 (76.5)	39 (79.6)	510 (77.4)	0.93	
Race/ethnicity, N (%)				0.02^b^	n.s.
White (non-Hispanic)	20 (58.8)	28 (57.1)	272 (41.3)		
Hispanic	2 (5.9)	5 (10.2)	74 (11.2)		
Black	11 (32.4)	15 (30.6)	296 (44.9)		
Asian	0 (0.0)	0 (0.0)	1 (0.2)		
Other	1 (2.9)	1 (2.0)	16 (2.4)		
Education (years)	12.4 (2.6)	13.0 (3.0)	13.1 (2.5)	0.33	
WRAT-4 Reading SS, M (SD)	95.6 (12.5)	97.8 (14.7)	93.8 (14.9)	0.16	
Employed, N (%)	6 (17.7)	8 (16.3)	232 (35.2)	< 0.01	3 > 1, 2
*Comorbidities*					
Hepatitis C, N (%)	12 (35.3)	11 (22.5)	153 (23.2)	0.30	
Hypertension, N (%)	6 (17.7)	8 (16.3)	108 (16.4)	0.98	
Hyperlipidemia, N (%)	3 (8.8)	5 (10.2)	53 (8.0)	0.87	
Diabetes, N (%)	3 (8.8)	6 (12.2)	49 (7.4)	0.51^c^	
CPD, N (%)	8 (23.5)	5 (10.2)	49 (7.4)	< 0.01^c^	1 > 3
Current smoker, N (%)	14 (41.2)	22 (44.9)	206 (31.3)	0.09	
Past smoker, N (%)	29 (85.3)	40 (81.6)	502 (76.3)	0.32	
*Psychiatric characteristics*					
Depressive sx (BDI-II)	25 [17.8–32.5]	22 [13.5–30.5]	9 [4–18]	< 0.01^d^	1, 2 > 3
Current MDD, N (%)	9 (26.5)	17 (34.7)	77 (11.8)	< 0.01	2, 1 > 3
LT MDD, N (%)	25 (73.5)	34 (69.4)	311 (47.4)	< 0.01	1, 2 > 3
Current substance use dx, N (%)	0 (0.0)	4 (8.2)	52 (7.9)	0.24^c^	
LT substance use dx, N (%)	25 (73.5)	35 (71.4)	469 (71.5)	0.97	
*HIV characteristics*					
Current CD4 (c/µL)	499.5 [333.5-554.5]	495 [251.5-710.5]	430 [276-625.8]	0.55^d^	
Nadir CD4 (c/µL)	119 [15.3-296.3]	203 [63.5–303]	180 [50–315]	0.62^d^	
HIV duration (years)	10.7 (6.8)	11.4 (5.7)	9.5 (6.5)	0.08	
On ART, N (%)	30 (88.2)	36 (73.5)	455 (69.0)	0.03	1 > 3
AIDS status, N (% AIDS)	21 (61.8)	32 (65.3)	386 (58.6)	0.62	
Plasma viral load, N (% ≤ 50 c/µL)	19 (55.9)	21 (43.8)	285 (43.9)	0.39	
*Neurocognition*					
Neurocognitive impairment, N (%)	13 (38.2)	14 (28.6)	204 (31.0)	0.63	
Global deficit score, mdn [IQR]	0.4 [0.3–0.9]	0.3 [0.1–0.7]	0.3 [0.1–0.6]	0.04^d^	1 > 3
Verbal fluency impairment, N (%)	7 (20.6)	11 (22.4)	89 (13.5)	0.16	
Executive function impairment, N (%)	8 (23.5)	11 (22.4)	143 (21.7)	0.96	
Processing speed impairment, N (%)	8 (23.5)	11 (22.4)	109 (16.5)	0.37	
Learning impairment, N (%)	17 (50.0)	17 (34.7)	230 (34.9)	0.21	
Memory impairment, N (%)	15 (44.1)	14 (28.6)	177 (26.9)	0.11	
Working memory impairment, N (%)	10 (29.4)	12 (24.5)	154 (23.4)	0.73	
Motor skills impairment, N (%)	14 (41.2)	18 (36.7)	107 (16.4)	< 0.01	1, 2 > 3
*Daily functioning*,* mdn [IQR]*					
IADL declines	7.5 [5–9]	4 [3–6]	1 [0–2]	< 0.01^d^	1 > 2 > 3
PAOFI complaints	15 [5.8–23.5]	11 [5–22]	2 [0–6]	< 0.01^d^	2, 1 > 3
KPS scale	80 [70–90]	80 [60–90]	90 [80–100]	< 0.01^d^	3 > 2, 1

Note. a = *p*-values are Benjamini-Hochberg adjusted using a False Discovery Rate (FDR) of 5%, b = White vs. non-White, c = Fisher’s exact test conducted as more than 20% of cells had expected frequencies < 5, d = Kruskal-Wallis test conducted due to non-normally distributed variable. SS = standard score, CPD = chronic pulmonary disease, BDI-II = Beck Depression Inventory-II, sx = symptoms, LT = lifetime, MDD = Major Depressive Disorder, ART = antiretroviral therapy, IADL = independent activities of daily living, PAOFI = Patient’s Assessment of Functioning Inventory, KPS = Karnofsky Performance Status. M = mean, SD = standard deviation, mdn = median, IQR = interquartile range

The final multinomial model with odds of being classified as an impaired functioning class (i.e., Classes 1_Impaired&Improving_ and 2_Impaired&Worsening_) versus Class 3_Unimpaired&Stable_ is shown in Table 5. Baseline depressive symptoms increased odds of Class 1_Impaired&Improving_ by 10% and Class 2_Impaired&Worsening_ by 8% for each additional depressive symptom (small effect sizes [87]). Motor skills impairment increased odds of Class 1_Impaired&Improving_ by 50% and Class 2_Impaired&Worsening_ by 59% for each point increase in deficit score (small effect sizes [87]). The odds of Class 1_Impaired&Improving_ were 3.79 times higher for PWH with CPD than PWH without (medium effect size [87]). Each additional year in age uniquely increased odds of Class 2_Impaired&Worsening_ by 6% (small effect size [87]).

**Table 5:** Final multinomial logistic regression predicting odds of impaired functioning trajectories

*N* = 734	Class 1 Impaired & Improving			Class 2 Impaired & Worsening		
Predictor	OR	95% CI	*p*-value	OR	95% CI	*p*-value
Age (years)	1.02	0.98–1.07	0.397	1.06	1.02–1.10	**0.004**
Depressive symptoms (BDI-II)	1.10	1.07–1.14	**< 0.001**	1.08	1.05–1.11	**< 0.001**
On ART (ref. off ART)	2.73	0.91–8.21	0.074	0.92	0.46–1.87	0.825
Employed (ref. unemployed)	0.68	0.26–1.74	0.417	0.50	0.22–1.14	0.098
Motor deficit score	1.50	1.04–2.14	**0.028**	1.59	1.18–2.14	**0.003**
CPD (ref. no CPD)	3.79	1.48–9.70	**0.005**	1.20	0.42–3.42	0.737

Note. Final predictors retained in the multinomial logistic regression after performing backwards stepwise selection. Odds ratios compare odds of impaired versus unimpaired trajectory classification. Overall model fit statistics: BIC = 612.0, AICc = 548.2, $\:{\chi\:}^{2}$(12) = 110.82, *p* < 0.01, Nagelkerke R^2^ = 0.24, OR = odds ratio, CI = confidence interval. *p*-values < 0.05 are bolded and denote statistical significance. BDI-II = Beck Depression Inventory-II; CPD = chronic pulmonary disease

### Changes in Global Cognition and Trajectory Class Membership

Participants at their final, 24 month visit (total *N* = 306; Class 1 *n* = 18, Class 2 *n* = 27, Class 3 *n* = 261) significantly differed in both total ($\:{\chi\:}^{2}$ = 11.39, *p* < 0.01) and average sRCS ($\:{\chi\:}^{2}$ = 11.66, *p* < 0.01) by class. Adjusted pairwise comparisons showed that Class 2_Impaired&Worsening_ (total sRCS: median = -0.59[IQR -1.54, -0.33]; average sRCS: median = -0.15[IQR -0.51, -0.08]) had worsening cognition versus Class 3_Unimpaired&Stable_ (total sRCS: median = 0.06[IQR -0.86, 0.76]; average sRCS: median = 0.02[IQR -0.21, 0.18]), which had more stable cognition (*p*-values < 0.02). Class 1_Impaired&Improving_ exhibited the greatest cognitive improvement on both total and average sRCS (median = 0.12[IQR -0.79, 1.17]; median = 0.04[IQR -0.20, 0.32], respectively); however, did not significantly differ from either Class 2_Impaired&Worsening_ or Class 3_Unimpaired&Stable_.

## Discussion

### Key Findings

The current study identified three everyday functioning trajectories over five semi-annual visits of follow-up. The majority of PWH (88%) had minimal functional declines at baseline with stable functioning across time. Two, small classes of PWH had elevated functional declines at baseline with differing change trajectories: worsening (7%) versus improvement (5%). Greater depressive symptoms and motor skills impairment were predictors of both impaired functioning classes, and older age predicted worsening functioning. Interestingly, having CPD predicted improvement in a group with functional impairment at baseline.

The baseline rate of functional dependence in the current sample (42%) defined by at least 2 IADL declines was in the range of previously reported rates (17–55%) [16, 17, 21, 88–92]. As found in prior U.S. studies of everyday functioning trajectories in older PWoH [49–55], three classes were identified with the largest class consisting of individuals with unimpaired and stable functioning. Like PWoH, most PWH did not exhibit significant functional decline, despite being at elevated risk for NCI. Consistent with past work, predictors of these trajectories included age, cognition, and comorbidities, but not other sociodemographic factors [51, 53]. The identification of an improving everyday functioning trajectory in this study is novel and promising. This class, though small, reported the highest number of functional declines at baseline, but exhibited a notable decrease in functional declines from 7 to 3 after two years.

The impaired and worsening functioning class was small relative to past studies of PWoH, which may be partially explained by the shorter time span, smaller sample size, and younger age range of the current study. Participants ranged in age from 18 to 69 years at baseline and half of participants were ages 38–49 years. After two years (i.e., end of study), only 6% of participants were at least 60 years old. Despite these limitations, age had a small significant effect on increasing odds of the impaired and worsening functioning class, but not in the impaired and improving functioning class. Proposed theories of premature and accelerated aging [7, 8, 93, 94] would support that age-related functional declines are observed at younger ages among PWH, though this could not be determined in the current study without a sample of PWoH. Though older age is associated with greater comorbidity burden and physical challenges [49], biological age may be a more precise predictor of disability than chronological age [50, 95, 96] and should be further explored in future work.

### Predictors of Impaired Functioning Trajectories

Motor skills impairment increased risk of both impaired everyday functioning trajectories. At baseline, PWH in the impaired functioning classes had higher rates of motor skills impairment (41% and 37%, respectively) relative to the unimpaired and stable class (16%). This finding supports that motor deficits persist in the post-ART era and is associated with greater risk of functional dependence [14, 27, 28]. Motor dysfunction among PWH is common and can be associated with basal ganglia atrophy [97–99], brain structures highly vulnerable to HIV. Alternatively, motor skills impairment may also be reflective of greater inflammation [100], neuropathic pain [101], and age-related comorbidities. For example, one study of PWH [102] found that though global cognition was stable over five years, motor function declined in the context of comorbidities such as cerebrovascular disease.

It was unsurprising that global cognition paralleled everyday functioning trajectories considering the robust evidence linking change in cognition to change in everyday functioning [42, 55, 56, 59]. Though less efficient than younger adult comparisons, older adults can exhibit considerable learning gains through motor skills training [103, 104] demonstrated by more rapid and accurate performance on tasks involving motor sequence learning (i.e., series of stereotyped movements) and motor adaptation (i.e., corrective responses to sensorimotor perturbations). The discrepancy in performance by age may be associated with age-related cognitive decline in domains such as processing speed and working memory, in addition to poorer memory consolidation [104]. Thus, benefits from cognitive interventions may also transfer to everyday functioning [105–107].

Greater depressive symptoms increased risk of everyday functioning impairment, in line with prior work [17, 19, 22, 36, 37]. Lifetime MDD was not retained in the final model, suggesting that depressive symptoms, which measured depression severity in closer temporal proximity to IADL declines, were a more reliable predictor of functional decline. It is worth noting that depression [22, 36] and fatigue [108] can negatively bias self-reported functional abilities in the absence of impairment on objective performance-based measures. Nonetheless, a CHARTER study [109] of biopsychosocial phenotypes found that 59% of a phenotype of depressed PWH with self-reported functional dependence exhibited NCI, suggesting that depression can contribute to objective impairments. Depression is also associated with psychomotor slowing [35, 110], which was observed in this study by a weak, but significant positive correlation. As such, targeting depressive symptoms coupled with good health behaviors (e.g., adherence) may result in optimal everyday functioning outcomes and improved motor functioning.

Though multiple medical comorbidities were considered for inclusion, only baseline rates of CPD differed across everyday functioning classes. Having CPD uniquely increased odds by nearly four-fold for the impaired and improving class, which exhibited the highest self-reported functional declines at baseline. Consistent with these findings, studies of older PWH and PWoH have shown that having a lung problem or disease differentiated everyday functioning trajectories [53] above other medical comorbidities (e.g., arthritis, diabetes) [51]. CHARTER studies [111, 112] of PWH have found that having CPD is associated with worsening cognition over time; however, those treated by a bronchodilator had better cognition. Likewise, PWH in the impaired and improving class may have experienced improvement due to treatment via connection to care. Though a substantial proportion (24%) of PWH in this class had CPD, this statistic only included 8 individuals due to the small class sample size. This finding should be further explored especially as the prevalence of CPD will likely increase among aging PWH due to longer exposure to HIV and smoking, and having both HIV and COPD is associated with greater physical limitations than either condition alone [113, 114].

The only HIV-disease characteristic that differentiated everyday functioning classes was ART status (on vs. off), in which the impaired and improving class unexpectedly had higher rates than the unimpaired and stable class. Though ART status was no longer a significant predictor after adjusting for other covariates, higher rates of ART in this class further supports our hypothesis that this class may experience better connection to care, which portends improved health outcomes. Though most of the sample exhibited unimpaired and stable functioning, the overall sample had generally lower rates of being on ART (70%). Some studies similarly did not find that HIV-disease characteristics predicted everyday functioning [48, 65], though this relationship may depend on cohort-specific effects [19] (e.g., nadir CD4 may not be a predictor of everyday functioning in PWH who initiate less neurotoxic ART earlier).

### Limitations

CHARTER did not include PWoH; thus, this study could not determine if everyday functioning trajectories were premature, accelerated, and/or accentuated. Moreover, CHARTER did not include measures of psychological resilience [115] or additional social determinants of health, which may have contributed to improvement in everyday functioning. Analyses were limited to two years due to increasing rates of attrition; however, five visits were adequate for LGMM. The current sample had relatively well-controlled HIV and were healthy and functional enough to participate in research; thus, are not entirely representative of all PWH. Further, the primary outcome of functional decline was measured via a self-report questionnaire. Though convenient, low cost, and extensively used in research and clinical settings, self-report measures are vulnerable to inaccuracies such as recall bias, social desirability, and poor insight; thus, objective performance-based measures should be considered in future work [36]. Everyday functioning measures do not always adequately account for the subjective relevance of activities (e.g., participant’s finances have always been managed by their partner). Future studies should examine specific IADLs that may be more sensitive to functional decline [116]. Lastly, there was no available information on which participants were being treated for CPD (i.e., bronchodilators).

## Conclusions

This study’s findings have several clinical implications. While it is promising that most aging PWH exhibit unimpaired and stable everyday functioning, a minority of PWH do not. Some predictors for everyday functioning impairment are modifiable, which is critically important for those at risk for functional decline. Interventions that target depression (e.g., cognitive behavioral therapy [117]), motor skills [103, 104], and cognition more broadly [106, 107] may reduce risk for functional decline. This study uniquely identified a group of PWH with high rates of functional impairment at baseline that decreased over time, which may reflect better connection to care. Thus, strategies to improve healthcare retention (e.g., patient navigation [118]) may increase functional independence. Future longitudinal work should explore factors (e.g., psychological resilience) that promote improvement in everyday functioning.

## Data Availability

Contact corresponding author.
